# Prevalence of GII.4 Sydney Norovirus Strains and Associated Factors of Acute Gastroenteritis in Children: 2019/2020 Season in Guangzhou, China

**DOI:** 10.1007/s12560-021-09482-0

**Published:** 2021-06-21

**Authors:** Lei Duan, Xiaohan Yang, Jia Xie, Wenli Zhan, Changbin Zhang, Hong Liu, Mengru Wei, Yuan Tang, Hongyu Zhao, Mingyong Luo

**Affiliations:** 1 Medical Genetic Centre, Guangdong Women and Children’s Hospital, Guangzhou Medical University, Guangzhou, 511442 People’s Republic of China; 2 Medical Genetic Centre, Guangdong Women and Children Hospital, Guangzhou, 511442 People’s Republic of China; 3 Department of Paediatrics, Guangdong Women and Children Hospital, Guangzhou, 511442 People’s Republic of China

**Keywords:** Norovirus, GII.4 Sydney, Epidemiological characteristics, Acute gastroenteritis, Winter

## Abstract

Norovirus, the leading cause of non-bacterial acute gastroenteritis (AGE) worldwide, is constantly mutating. Continuous monitoring of the evolution of epidemic genotypes and emergence of novel genotypes is, therefore, necessary. This study determined the prevalence and clinical characteristics of norovirus strains in AGE in Guangzhou, China in 2019/2020 season. This study included children aged 2–60 months diagnosed with AGE in Guangzhou Women and Children Hospital, from August 2019 to January 2020. Norovirus was detected by real-time polymerase chain reaction and clinical data were obtained. Genotyping and phylogenetic analyses were performed with partial gene sequence fragments located within the open reading frames 1 and 2. During the study period, 168 children (61.3% males) were confirmed as norovirus infectious AGE. The main symptoms were diarrhoea and vomiting and 38 patients (22.6%) had seizures. Norovirus was mainly prevalent in October and November, and GII.4 Sydney[P31] was the major genotype circulating in Guangzhou. The phylogenetic tree showed that the Guangzhou strains had high homology with the strains circulating in 2017–2019 worldwide. GII.4 Sydney was the main prevalent norovirus genotype in Guangzhou from August 2019 to January 2020, which had more severe diarrhoea than those of other genotypes. These findings provide a valuable reference for the prevention, control, and treatment of norovirus in the future.

## Introduction

Norovirus is the leading cause of non-bacterial acute gastroenteritis (AGE) worldwide, causing approximately 18% of AGE and 212,000 deaths every year (Ahmed et al., 2014; Pires et al., 2015). Norovirus causes considerable yearly losses to society, and more than half of the expenditure on norovirus is due to childhood infections (Bartsch et al., 2016). Norovirus is mainly prevalent in winter and often causes severe AGE outbreaks. Norovirus has a strong transmission capacity and is mainly transmitted from person to person and foodborne. The symptoms of patients with norovirus AGE mainly include vomiting, diarrhoea, nausea, etc. Most of these symptoms are mild and self-limiting and generally last for 2–3 days. However, norovirus can cause serious clinical symptoms in some groups, especially children, the elderly and immunocompromised patients (Green, 2014; O'Brien et al., 2016). Once transmitted, the virus can remain in the patient for weeks or even a year among the immunocompromised (Ludwig et al., 2008). Due to the lack of specific antiviral drugs and vaccines, supportive care and symptomatic treatment are the main therapeutic methods for norovirus infections. Moreover, norovirus has a strong ability to spread and usually causes AGE outbreaks in semi-enclosed areas, such as hospitals, schools, kindergartens, cruise ships, nursing homes and prisons (Ahmed et al., 2014; Wang et al. 2016b).

Norovirus is a 7.5–7.7 kb long single-stranded positive-sense RNA virus belonging to the family *Caliciviridae* and its RNA includes three open reading frames (ORFs). Norovirus is divided into 10 genogroups (GI to GX, but only GI, GII and GIV can infect humans) based on the ORF2 gene and further divided into more than 40 genotypes (Chhabra et al., 2019). Because the recombination of norovirus mainly occurs at the ORF1 and ORF2 junctions, a dual typing based on both the RdRp of ORF1 gene (P type) and the VP1 of ORF2 gene (genotype) of norovirus has been widely used since 2013 (Kroneman et al., 2013). GII.4 has been the most popular genotype worldwide, accounting for more than 90% of all the epidemics (Bull & White, 2011; Chhabra et al., 2019). GII.4 can be further classified into eight strains due to its continuous variations and the novel GII.4 epidemic strains usually emerge every 2–3 years (Cannon et al., 2017; Wang et al. 2019b). In China, norovirus is also a major cause of non-bacterial AGE and causes more than 20% AGE nationwide and affects about 800,000 people yearly (Zhou et al., 2017). In recent years, some studies have shown that non-GII.4 (such as GII.17 and GII.2) have gradually replaced GII.4 to become the predominant genotypes (Ao et al., 2018; Jin et al., 2020). Continuous monitoring of the evolution of epidemic genotypes and the emergence of novel genotypes is necessary to effectively control norovirus transmission.

In this study, we aimed to analyse the epidemiological and clinical characteristics of norovirus infection in children in Guangzhou in the 2019/2020 season. Furthermore, we performed a phylogenetic analysis of the epidemic strains prevalent in Guangzhou to understand any trends of norovirus.

## Materials and Methods

### Study Population and Specimen Collection

A total of 417 children (age range: 2–60 months) with clinical diagnoses of AGE at Guangdong Women and Children Hospital, from August 2019 to January 2020, were included in our study. All norovirus infection diagnoses were confirmed with molecular detection (described below) at the clinical laboratory. AGE was defined as defaecation ≥ 3 times within 24 h with trait changes (diluted watery stools) and/or vomiting ≥ 2 times within 24 h (Liao et al., 2016).

### Collection of Clinical Information

Demographic characteristics and clinical symptoms were collected from the participants’ medical records. Laboratory examinations, mainly including hematological and biochemical parameters, were also collected. The severity of AGE was assessed by the Modified Vesikari Score (MVS) System (Wang et al. 2016a), and it was defined as 0–8, mild; 9–10, moderate; ≥ 11, severe. This study did not record any data about the patient’s personal identity information since the collection process started and all patient names and other relative information were replaced with identifying numbers. Therefore, the informed consent was not needed. The study was approved by the Ethics Committee of Guangdong Women and Children Hospital (ref.202001189).

### Norovirus RNA Extraction and Detection

Norovirus RNA was extracted from stool and vomit samples using an RNA Extraction Kit (Tianlong, Xi’an, China) based on magnetic beads, according to the manufacturer’s instructions. Norovirus RNA was detected using Diagnostic Kit for Norovirus RNA (PCR Fluorescence Probing) (Land medical, Wuhan, China) on the ABI 7500 Fast Real-Time polymerase chain reaction (PCR) platform (Applied Biosystems, Foster, USA), and positive samples were further sequenced and typed.

### Genotyping and Phylogenetic Analyses

Reverse transcription was performed using PrimeScript™ RT reagent Kit with gDNA Eraser (Takara, Beijing, China) to obtain norovirus cDNA. Norovirus belonging to genotypes GI and GII were amplified using primers MON432 and GISKF, MON431 and GIISKR, respectively (Cannon et al., 2017). The length of the PCR-amplified products was expected to be 543 bp for GI and 557 bp for GII, including the partial RdRp gene located in ORF1 as well as the partial VP1 gene located in ORF2 (ORF1-2 junction) (Kroneman et al., 2013). The amplified products were sent to Sangon Biotech (Sangon Biotech, Shanghai, China) for sequencing. To confirm the genotype of norovirus and to analyse the norovirus sequences at the ORF1-2 junction, we used the online Norovirus Typing Tool Version 2.0 (http://www.rivm.nl/mpf/norovirus/typingtool). The sequences were uploaded to GenBank (accession numbers: MT856488-MT856646).

Multiple sequence alignment was performed using ClustalW in MEGA X(Kumar et al., 2018), and the phylogenetic tree (based on the partial sequences of ORF1 and 2) was constructed using the neighbour-joining method, with a bootstrap value of 1000 repetitions. All reference sequences were downloaded from GenBank.

### Statistical Analysis

The data of patient’s clinical information were collated, imported and analysed with IBM SPSS Statistics for Windows, version 26.0 (IBM Corp., Armonk, N.Y., USA). Qualitative data were expressed as frequency (percentage), and the chi-square test was used for analysis. Quantitative data were expressed as the median and interquartile range (IQR), and t-test or one-way analysis of variance (ANOVA) was used to analyse data that conformed to the normal distribution, while the non-parametric rank-sum test was used for data that did not conform. Differences were defined as statistically significant when *p* < 0.05.

## Results

### Epidemiological Characteristics

**Fig. 1:**
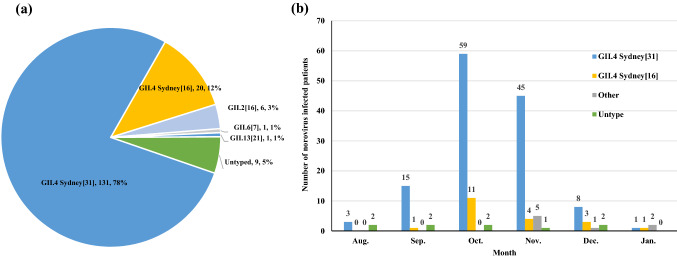
Distribution of norovirus genotypes associated with AGE in Guangzhou in 2019/2020 season. **a** Total distribution of norovirus genotypes in Guangzhou in 2019/2020 season. **b** Monthly distribution of the children with norovirus infectious AGE in Guangzhou in 2019/2020 season. The “Others” group included three genotypes: GII.2[P16], GII.6[P7] and GII.13[P21]

From August 2019 to January 2020 in Guangzhou, China, a total of 168 (40.29%) children were diagnosed with norovirus infection in this study (Fig. 1a). Among these patients, 159 were successfully genotyped, while 9 failed due to their low viral load unable to be sequenced. According to the RdRp gene on ORF1 and the VP1 gene on ORF2 (ORF 1–2 junctions), the majority of the norovirus genotypes were GII.4 Sydney[P31] and GII.4 Sydney[P16], in 131 and 20 AGE cases, respectively. In addition, there were 6, 1, and 1 case of GII.2[P16], GII.6[P7] and GII.13[P21], respectively.

As shown in Fig. 1b, the number of norovirus-infected children showed a monthly increase from 5 in August to 72 in October; in November, there were 55 infected children. The gradually decreasing trend started in November, with only four positive norovirus samples in January. GII.4 Sydney[P31] norovirus was the dominant genotype in Guangzhou, Guangdong Province, in the winter of 2019/2020. In October and November, GII.4 Sydney[P16] and GII.2[P16] also increased slightly.

### Demographic Characteristics and Clinical Manifestations

The demographic characteristics and clinical manifestations of the 168 children with norovirus infection in this study are shown in Table 1. Among these children, 103 (61.3%) were male and 45 (38.7%) were female. The median age was 19.0 (IQR 13.0–28.8) months, and 148 (88.1%) patients were under 3 years of age. A total of 80 (47.6%) children were hospitalised for a median of 5.0 (IQR 3.0–7.5) days. There were 56 (33.3%) children with fever, and the median body temperature during infection was 37.0 °C (IQR 37.0–37.9 °C). Eighty-two (48.8%) patients had anorexia and 38 (22.6%) had seizures. Clinical data showed that the main symptoms shown by the patients with norovirus infection were diarrhoea (79%) and vomiting (83.9%), and no significant differences were found between the genotype groups. However, compared with “Others” group, the clinical symptoms of “GII.4” group were more severe. “GII.4” group had a longer diarrhoea duration and a greater maximum number of diarrhoeal stools per day than those with “Others” group (both *p* < 0.05). Besides, the median MVS was higher among GII.4 infected children (10.0; IQR 8.0–13.0) compared with cases from “Others” group (8.0; IQR: 6.3–10.0); *p* < 0.05.

**Table 1:** Demographic characteristics and clinical manifestations of norovirus-infected patients

	Total	GII.4		Others^a^	Untyped
		GII.4 Sydney[P31]	GII. 4 Sydney[P16]		
Number of patients	168 (100.0%)	131 (78.0%)	20 (11.9%)	8 (4.8%)	9 (5.4%)
Sex(male)	103 (61.3%)	80 (61.1%)	12 (60.0%)	6 (75.0%)	5 (55.6%)
Age (month)	19.0 (13.0–28.8)	19.0 (13.0–28.0)	19.5 (14.3–38.5)	23.0 (21.3–36.8)	16.0 (11.5–29.0)
Number of hospitalisation	80 (47.6%)	63 (48.1%)	8 (40.0%)	3 (37.5%)	6 (66.7%)
Length of hospital stay(day)	5.0 (3.0–7.5)	5.0 (3.0–6.0)	4.5 (3.0–9.0)	8.0 (3.0–10.0)	9.5 (3.0–12.0)
Fever	56 (33.3%)	41 (31.3%)	9 (45.0%)	4 (50.0%)	2 (22.2%)
Body temperature (℃)	37 (37–37.9)	37 (37–37.8)	37 (37–38)	37.5 (37–39.73)	37 (36.6–37.4)
Vomiting	141 (83.9%)	111 (84.7%)	17 (85.0%)	7 (87.5%)	6 (66.7%)
Vomiting duration(day)	2.0 (1.0–3.0)	2.0 (1.0–3.0)	1.5 (1.0–3.0)	1 (1.0–1.8)	1 (0.0–3.5)
Max. no. vomiting episodes/24 h	3.0 (2.0–6.0)	3.0 (2.0–6.0)	4.0 (3.0–9.5)	4.0 (1.5–4.8)	3.0 (0.0–6.0)
Diarrhoea	132 (79.0%)	104 (80.0%)	15 (75.0%)	4 (50.0%)	9 (100.0%)
Diarrhoea duration (day)	2.0 (1.0–5.0)	2.0 (1.0–5.0)	2.0 (0.3–6.0)	0.5 (0.0–1.0)^b^	2.0 (1.0–3.0)
Max. no. diarrhoea stools/24 h	3.0 (1.0–6.0)	3.0 (1.0–5.0)	5.0 (0.3–8.8)	1.0 (0.0–2.8)^c^	4.0 (3.0–4.5)
Anorexia	82 (48.8%)	70 (53.4%)	8 (40.0%)	2 (25.0%)	2 (22.2%)
Dehydration	15 (8.9%)	13 (9.9%)	1 (5.0%)	1 (12.5%)	0 (0.0%)
Abdominal pain	22 (13.1%)	15 (11.5%)	4 (20.0%)	2 (25.0%)	1 (11.1%)
Bloating	11 (6.5%)	10 (7.6%)	1 (5.0%)	0 (0.0%)	0 (0.0%)
Nausea	21 (12.5%)	15 (11.5%)	3 (15.0%)	1 (12.5%)	2 (22.2%)
Seizures	38 (22.6%)	30 (22.9%)	5 (25.0%)	1 (12.5%)	2 (22.2%)
Modified Vesikari scale (MVS)	10.0 (8.0–13.0)	10.0 (8.0–13.0)	10.0 (8.0–14.5)	8.0 (6.3–10.0)^d^	9.0 (7.0–13.0)

^a^The “Others” group contained three genotypes: GII.2[P16], GII.6[P7] and GII.13[P21]

^b^*p* = 0.004 < 0.05: GII.4 (2.0; IQR: 1.0–5.0) vs. Others

^c^*p* = 0.011 < 0.05: GII.4 (4.0; IQR: 1.0–6.0) vs. Others

^d^*p* = 0.044 < 0.05: GII.4 (10.0; IQR: 8.0–13.0) vs. Others

### Laboratory Examinations

Laboratory examinations showed that the white blood cell (WBC), red blood cell (RBC), aspartate aminotransferase (AST), hydroxybutyrate dehydrogenase (HBDH) and creatine kinase isoenzyme (CK-MB) levels in the blood of patients with norovirus infection increased, but no significant differences were found between the genotype groups (Table 2). The levels of sodium (Na^+^) and chloride (Cl^−^) in the blood of patients infected with GII.4 were significantly lower than those in the “Others” (both *p* < 0.05).

**Table 2:** Laboratory examinations of patients associated with the different norovirus genotypes

	Total	GII.4		Others^a^	Untype
		GII.4 Sydney[P31]	GII. 4 Sydney [P16]		
Total number of patients	168 (100.0%)	131 (78.0%)	20 (11.9%)	8 (4.8%)	9 (5.4%)
hs-CRP (0–10 mg/L)	1.3 (0.4–5.0)	1.2 (0.3–5.1)	2.7 (0.4–5.4)	1.1 (0.5–6.2)	1.2 (0.5–9.5)
WBC (3.5–9.5 × 109/L)	9.6 (7.0–12.8)	9.67 (6.9–13.4)	10.2 (7.5–12.1)	12.38 (8.1–20.1)	7.4 (6.8–9.3)
NEUT (1.8–6.3 × 10^9^/L)	5.1 (2.64–9.21)	4.9 (2.6–9.3)	5.4 (2.8–8.1)	9.7 (4.0–17.9)	4.0 (1.2–6.7)
LYMPH (1.1–3.2 × 10^9^/L)	3.0 (1.9–4.5)	2.9 (1.9–4.6)	3.9 (2.9–4.3)	2.5 (0.9–5.6)	2.1 (1.4–4.2)
RBC (4.0–4.5 × 10^9^/L)	4.7 (4.5–4.9)	4.7 (4.5–4.9)	4.6 (4.4–4.9)	5.1 (4.8–5.2)	4.65 (4.6–4.9)
PLT (125–350 × 10^9^/L)	325 (272.5–392.5)	332 (273.5–400.0)	314 (268–438.3)	314 (202.0–367.0)	329.0 (234.0–368.0)
Na^+^ (137–147 mmol/L)	135.9 (134.4–137.6)	136.0 (134.3–137.6)	134.7 (132.6–135.6)	139.2 (137.1–140.7)^b^	135.55 (133.7–137.3)
Cl^−^ (99–110 mmol/L)	102.25 (100.3–105.0)	103 (99.7–105.3)	102.3 (99.9–105.1)	105.5 (104.9–107.1)^c^	101.7 (97.4–106.2)
LDH (180–430 U/L)	410.4 (310.7–472.2)	413.6 (329.4–488.7)	412 (337.9–467.0)	340.2 (291.1–427.9)	294.5 (234.9–411.4)
AST (15–40 U/L)	41.0 (34.0–48.0)	41.0 (34.3–48.0)	39.0 (31.5–51.0)	35 (25.3–60.5)	40.5 (30.3–59.0)
HBDH (72–182 U/L)	226.0 (197.0–249.5)	229.0 (208.5–259.0)	216.0 (184.3–241.0)	222.5 (166.0–262.5)	200.0 (182.8–233.0)
CK-MB (0–25 U/L)	39.0 (29.0–47.0)	40.0 (30.5–47.0)	35.0 (22.0–63.5)	27.5 (20.0–86.5)	44.5 (40.3–45.8)
FOBT	24 (14.3%)	20 (15.3%)	2 (10.0%)	2 (25.0%)	0 (0.0%)

*hs-CRP* high-sensitivity C-reactive protein, *WBC* white blood cell, *NEUT* neutrophils, *LYMPH* lymphocytes, *RBC* red blood cell, *PLT* platelets, *Na* sodium, *Cl* chloride, *LDH* lactate dehydrogenase, *AST* aspartate aminotransferase *HBDH* hydroxybutyrate dehydrogenase, *CK-MB* creatine kinase isoenzyme, *FOBT* foecal occult blood test

^a^The “Others” group contained three genotypes: GII.2[P16], GII.6[P7] and GII.13[P21]

^b^*p* = 0.003 < 0.05: GII.4 (135.8; IQR: 134.2–137.4) vs. Others

^c^*p* = 0.007 < 0.05: GII.4 (102.3; IQR: 99.9–105.1) vs. Others

### Phylogenetic Analysis

**Fig. 2:**
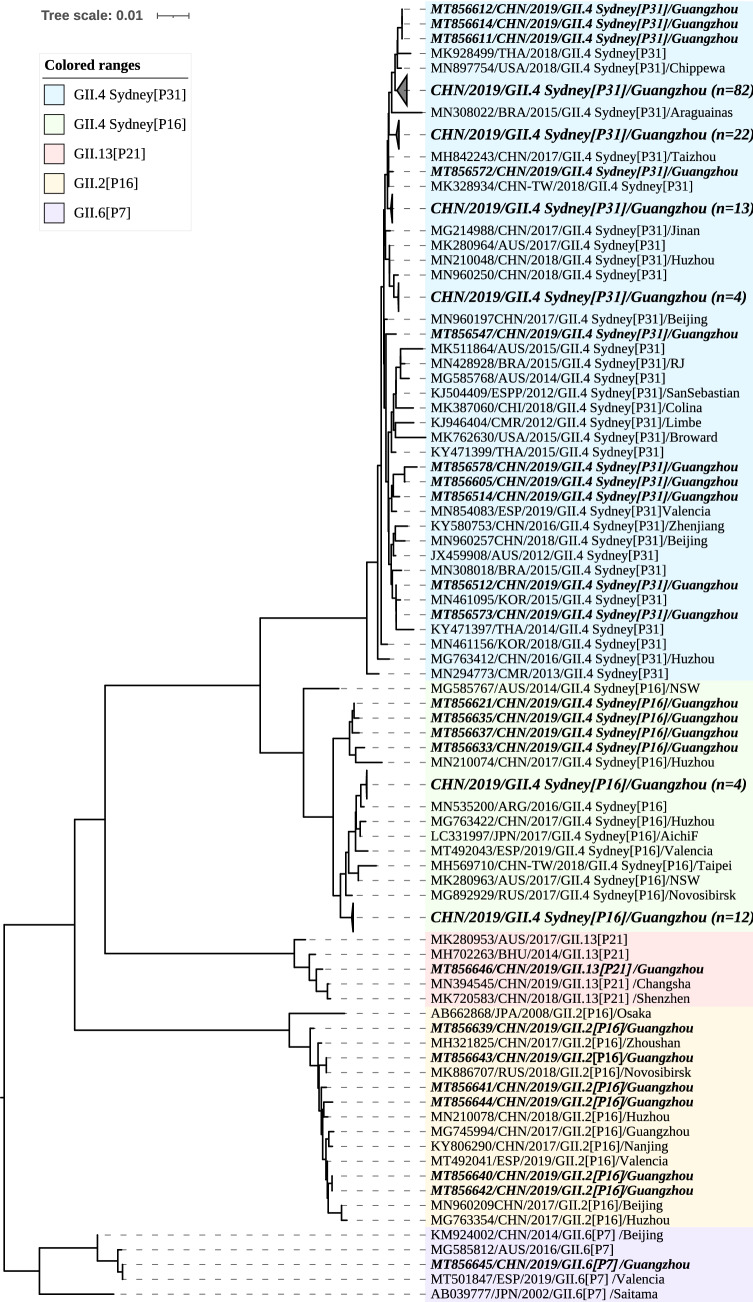
Phylogenetic analysis of the 445 bp norovirus nucleotide sequence within the partial RdRp and VP1 genes. The norovirus strains detected in this study (MT856488-MT856646) and reference sequence retrieved from GenBank were constructed using the neighbour-joining method with bootstrap analysis of 1000 replicates using MEGA X. The nucleotide sequence is presented as GenBank accession number/country/year of isolation/genotype, and the sequences obtained in this study are indicated as bold italics

Phylogenetic analysis based on the partial gene fragment (ORF1/2 junction) showed the norovirus strains prevalent in Guangzhou in 2019/2020 season had high homology with the strains mainly circulated in China, Russia and the USA in recent years. The sequences alignment showed that 131 strains GII.4 Sydney[P31] shared 94.9–100.0% of the nucleotide identity level, as well as 97.7–99.5% nucleotide identities respect to the GII.4 Sydney[P31] prototype strain (GenBank accession no. JX459908). The closest relatives of these strains were several strains isolated in China, the USA and Thailand (MH842243, MH842243, and MK928499) with nucleotide identities of 97.0–99.8% (Fig. 2). Meanwhile, twenty strains GII.4 Sydney[P16] were closely related to viruses isolated from China, Argentina and Russia (MN210074, MN535200, and MG892929) between 2016 and 2018, with nucleotide identities of 96.1–99.6%, and they were displayed as 95.3–95.6% nucleotide identities compared to the prototype strain of GII.4 Sydney[P16] (MG585767). In addition, 6 strains GII.2[P16] were found in this study, and they shared 95.3–95.8% of nucleotide identities with the prototype strain of GII.2[P16] (AB662868). Phylogenetic analysis showed that the 6 strains were highly homologous to the strains from China, Russia and Spain (MH321825, MK886707 and MT492041) in 2017 and 2018, with nucleotide identities of 98.9–100.0%.

**Fig. 3:**
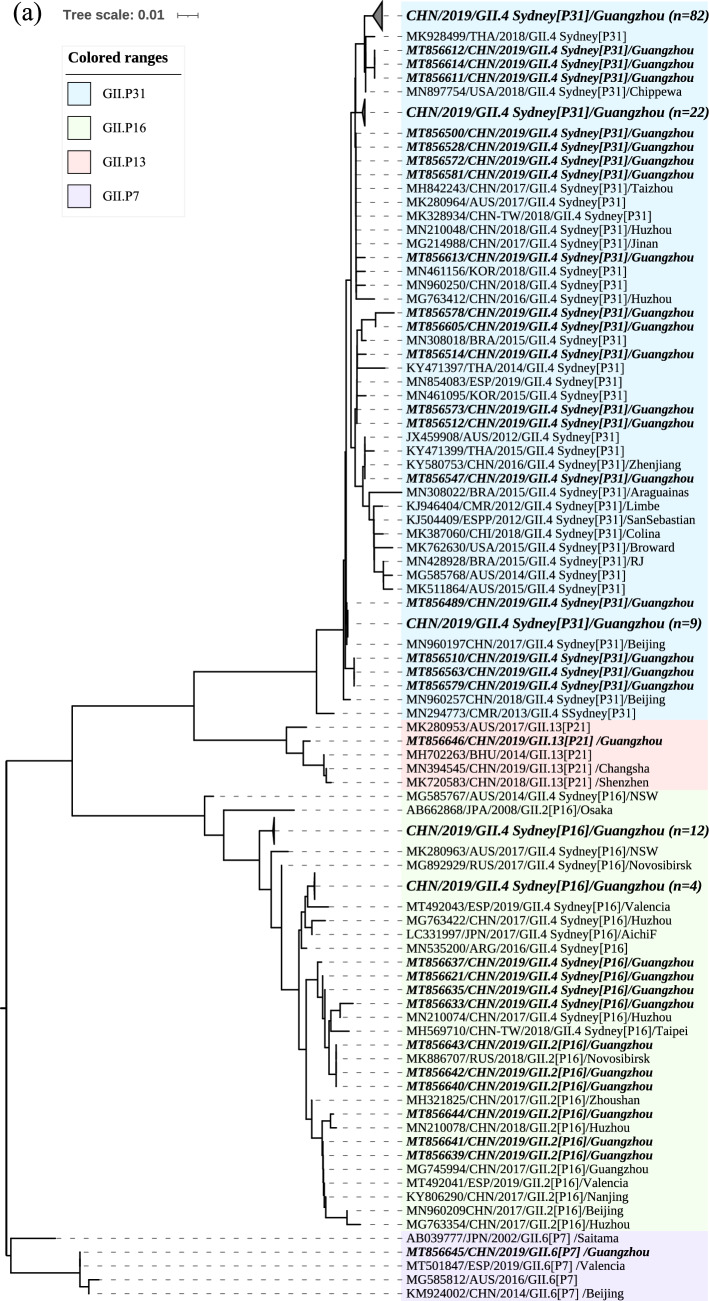

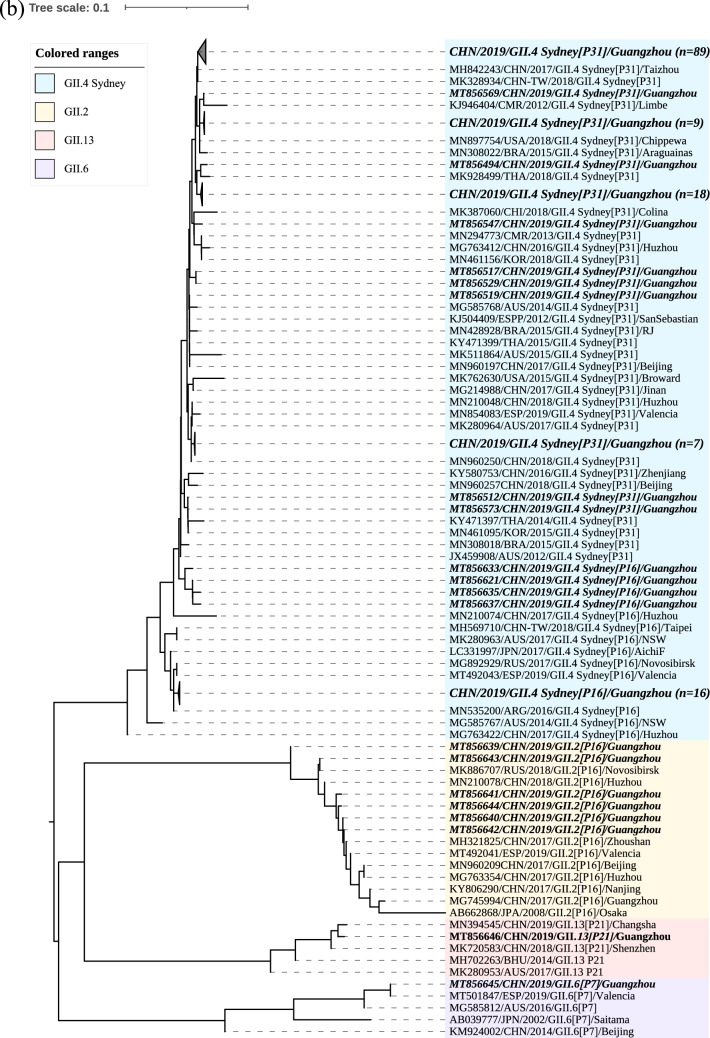
Phylogenetic analysis based on **a** the partial RdRp genes(228 bp) and **b** the partial VP1 genes (237 bp). The norovirus strains detected in this study (MT856488–MT856646) and reference sequence retrieved from GenBank were constructed using the neighbour-joining method with bootstrap analysis of 1000 replicates using MEGA X. The nucleotide sequence is presented as GenBank accession number/country/year of isolation/genotype, and the sequences obtained in this study are indicated as bold italics

To analyse the phylogenetic characteristics of the recombinant strains GII.4 Sydney[P16], phylogenetic trees based on the partial RdRp sequence (228 bp) and the partial VP1 sequence (237 bp) were also constructed, respectively. As shown in Fig. 3a, the nucleotide identities between 20 recombinant strains GII.4 Sydney[P16] and 6 strains GII.2[P16] were 96.4–99.6% based on RdRp gene. The recombinant strains GII.4 Sydney[P16] in our study showed high homology with the strains from China, Argentina and Russia (MN210074, MN535200, and MG892929) with nucleotide identities of 97.3–99.6%. For VP1 gene, the nucleotide identities between 20 recombinant strains GII.4 Sydney[P16] and 131 strains GII.4 Sydney[P31] were 93.3–98.7%. The recombinant strains GII.4 Sydney[P16] in our study showed high homology with the strains from China, Argentina and Russia with nucleotide identities of 94.3–100.0% (Fig. 3b).

## Discussion

In this study, we reported the detailed clinical and epidemiological characteristics of young children with norovirus AGE in Guangzhou City in the 2019/2020 season. Our results revealed that norovirus was prevalent mainly in October and November during our study period, and GII.4 Sydney[P31] was the major genotype circulating in Guangzhou, followed by GII.4 Sydney[P16] and GII.2[P16]. Additionally, diarrhoea and vomiting were the main symptoms of norovirus AGE and GII.4 Sydney was associated with a more severe AGE presentation than other genotypes. These findings provide a better understanding of the clinical manifestations and aetiology of norovirus AGE, and can be helpful in the prevention, control and treatment of norovirus AGE in the future.

GII.4 Sydney[P31] was the main epidemic strain (78.0%) in Guangzhou in 2019/2020 season in our study. This strain was first detected in Australia in 2012 and rapidly became the major epidemic strain in Europe, America and Asia in 2012–2013 (Leshem et al., 2013; van Beek et al., 2013). In 2015, a novel recombinant norovirus strain, GII.4 Sydney[P16], appeared and prevailed in Germany, France, the USA and China (Bidalot et al., 2017; Ruis et al., 2017; Xue et al., 2019). There were 20 cases (11.9%) of GII.4 Sydney[P16] during the study period. Previous studies have found that the RdRp gene of this novel strain is highly homologous to GII.2[P16], and the VP1 gene is highly homologous to GII.4 Sydney[P31] (Arias et al., 2016; Lun et al., 2018). In addition, a few strains of GII.2[P16], GII.6[P7] and GII.13[P21] were also found in our study. GII.2[P16] was the major epidemic strain in Guangdong Province in August 2016 and continued to circulate in 2017(Ao et al., 2018). The main epidemic strains were GII.4 Sydney[P31] (78.0%) and GII.4 Sydney[P16] (11.9%) in this study, which was different from previous years in Guangzhou(Wang et al. 2019a). This suggests that the norovirus epidemic trend may have changed in Guangzhou in the 2019/2020 season, but further studies are needed to verify it.

According to the norovirus surveillance network in China, more than 80% of norovirus outbreaks occurred during the winter season (November to March) from 2006 to 2018 (Jin et al., 2020; Qin et al., 2017; Wang et al. 2019a). However, our data revealed that the majority (75.60%) of norovirus cases presented from October and November in Guangzhou in 2019/2020 season, which was slightly earlier than the previous data of Guangzhou (Wang et al. 2019a). Marshall et al. revealed that meteorological factors may be able to play a role in the seasonal pattern of norovirus epidemics (Marshall & Bruggink, 2011). Based on the data from the Guangdong Meteorological Service, we found that from October 16 to December 31, 2019, the precipitation in Guangzhou was 20.2 mm, 79% less than the same period in history (94.4 mm). Lack of rainfall and drought might be one of factors influenced the seasonal pattern of norovirus in Guangzhou in 2019. However, comprehensive studies from epidemiology, virology and meteorology are required to further understand the prevalence of norovirus.

In this study, male patients were more among the infected than females (103:65), which is consistent with previous studies (Ao et al., 2017; Wang et al. 2019a). That the innate and acquired immunity in females are inherently stronger than in males may be the reason (Jaillon et al., 2019). Children with norovirus infection are more likely to develop AGE, especially children under 5 years. In our study, more than 88% of the patients were aged < 36 months, with a median age of 19 (IQR 13–28.75) months. Fifty percent of infected patients were aged 12 to 23 months, which is consistent with previous reports(Mathew et al., 2019; Shioda et al., 2015; Zhirakovskaia et al., 2015). These results suggest that children under 3 years of age are more susceptible to norovirus infection. Therefore, in the prevention and control of norovirus, more attention should be paid to children in this age group.

Compared with non-GII.4, the clinical feature of GII.4-associated AGE is more severe, especially in diarrhoea and abdominal pain (Desai et al., 2012; Leshem et al., 2013). Our data indicated that “GII.4” group had a longer diarrhoea duration and a greater maximum number of diarrhoeal stools per day than those with “Others” group (both *p* < 0.05), which was consistent with previous studies (Haddadin et al., 2020; Wang et al. 2019a). The MVS has been used in several studies of norovirus infections to evaluate the severity of AGE (Bhavanam et al., 2020; Wang et al. 2016a). In this study, the MVS of “GII.4” group was statistically higher than “Others” group (10 vs. 8, *p* < 0.05), and this result was similar to a recent study (Bhavanam et al., 2020). In addition, the laboratory examination results showed that the levels of Na^+^ and Cl^−^ in the blood of patients with GII.4 infection were lower than those with other genotypes. All these features indicate that the GII.4 was more serious, and effective measures, such as rehydration, are needed to prevent more serious complications. Based on the RdRp gene, the GII.4 Sydney strain in this study can be divided into GII.4 Sydney[p31] (78.0%) and GII.4 Sydney[p16] (11.9%). The RdRp gene encodes a non-structural protein that participates in the transcription of norovirus, which can influence viral transmission (Arias et al., 2016; Ruis et al., 2017). However, our results showed no significant differences in epidemic and clinical characteristics were found between patients with GII.4 Sydney[P31] and GII.4 Sydney[P16] infection, which illustrated that recombination associated with RdRp in this study has few influences on the norovirus epidemic, but further studies are needed.

Our research showed nausea, abdominal pain and dehydration as the complications of norovirus infection. In addition, we recorded that 38 patients had seizures, accounting for 22.6% of the 168 norovirus-infected children in this study. The result was consistent with previous studies (Chen et al., 2009, 2019; Ma et al., 2019), which also showed that norovirus infection has a significantly higher incidence of seizures than rotavirus infection. Seizures, in this study, were similar to benign convulsions associated with mild gastroenteritis reported previously by Chen et al. (2018). The mechanism of norovirus-induced seizures in children is still unclear, and may be associated with the children’s immature immune system (Chen et al., 2019). Recently, some studies found that norovirus infection can cause encephalitis/encephalopathy (Chen et al., 2019; Sánchez-Fauquier et al., 2015). However, no case of encephalitis/encephalopathy was found in our study. In addition, the Laboratory examination data showed that the levels of cardiac biomarkers, such as HBDH and CK, were elevated in most children. This suggests the need for clinical attention to myocardial damage in patients with norovirus infection.

There were several limitations to our study. For example, only 6 months of norovirus epidemic data were collected during the 2019/2020 season, not a year-round study. The SARS-CoV-2 pandemic occurred in early 2020, and strict prevention and control policies were adopted in our country. The increase in people's hygiene awareness has led to a sharp drop in the number of norovirus infections, even after January there were no patients for a long time. In addition, we only analysed the epidemiological data of patients who visited the hospital, and patients who did not seek for medical advice at the hospital were not included in this study.

In conclusion, this study reported the epidemiological characteristics of norovirus from August 2019 to January 2020 in Guangzhou. The main genotypes of norovirus were GII.4 Sydney[P31] and GII.4 Sydney[P16]. Most of their strains had high homology with the epidemic strains of 2017–2019. We determined the clinical information of infected patients. Vomiting and diarrhoea were the main symptoms. These findings help us to understand the prevalence of norovirus strains in Guangzhou and provide a valuable reference for the prevention, control and treatment of norovirus in the future.

## Data Availability

The datasets generated and/or analysed during the current study are available in the GenBank repository under accession numbers MT856488-MT856646 (www.ncbi.nlm.nih.gov/sites/myncbi/1x7zkdP4afG5ea/collections/60686265/public/).

## Code Availability

IBM SPSS Statistics for Windows, version 26.0 (IBM Corp., Armonk, N.Y., USA).
